# Structure and dynamics of financial networks by feature ranking method

**DOI:** 10.1038/s41598-021-97100-1

**Published:** 2021-09-02

**Authors:** Mahmudul Islam Rakib, Ashadun Nobi, Jae Woo Lee

**Affiliations:** 1 Department of Computer Science and Telecommunication Engineering, Noakhali Science and Technology University, Sonapur, Noakhali 3814 Bangladesh; 2 Department of Physics, Inha University, Incheon, Republic of Korea

**Keywords:** Mathematics and computing, Physics

## Abstract

Much research has been done on time series of financial market in last two decades using linear and non-linear correlation of the returns of stocks. In this paper, we design a method of network reconstruction for the financial market by using the insights from machine learning tool. To do so, we analyze the time series of financial indices of S&P 500 around some financial crises from 1998 to 2012 by using feature ranking approach where we use the returns of stocks in a certain day to predict the feature ranks of the next day. We use two different feature ranking approaches—Random Forest and Gradient Boosting—to rank the importance of each node for predicting the returns of each other node, which produces the feature ranking matrix. To construct threshold network, we assign a threshold which is equal to mean of the feature ranking matrix. The dynamics of network topology in threshold networks constructed by new approach can identify the financial crises covered by the monitored time series. We observe that the most influential companies during global financial crisis were in the sector of energy and financial services while during European debt crisis, the companies are in the communication services. The Shannon entropy is calculated from the feature ranking which is seen to increase over time before market crash. The rise of entropy implies the influences of stocks to each other are becoming equal, can be used as a precursor of market crash. The technique of feature ranking can be an alternative way to infer more accurate network structure for financial market than existing methods, can be used for the development of the market.

## Introduction

The complex dynamic of financial market has been a place of interest for many researchers in last two decades^1–6^. Reconstructing or inferring an unknown network structure from the available monitored time series data has also been a foremost modern network science problem^7–9^. There are many approaches to analyze the time series of stocks for constructing the network. One of the approaches is the Pearson correlation of the returns of the stocks which has been used in last two decades^10^. In this approach, the threshold network is constructed assigning a threshold from the correlations of the stocks. The decision on the link presence or absence is based on pair-wise correlation between nodes. But the correlation between the given pair of nodes is linear. However, there can be also a nonlinear relationship between two stocks. To address this problem, the nonlinear correlation between stocks known as mutual information is also used to analyze the time series of financial indices in recent years^11–13^. Then, using mutual information, minimum spanning tree (MST), planar maximally filtered graph (PMFG) and also threshold networks are constructed and the network topologies are determined^14^. But mutual information takes univariate stance in correlation.

In this article, we use a new approach known as feature ranking of machine learning which can take a multivariate view on the correlation^15–17^. This approach was completely different than the existing correlation and mutual information technique. In recent past, this method has been used in logistic chaotic time series generated by using logistic map and to construct dynamic network^15^. Hence its real-world applicability was not checked. In this paper, we apply this approach in financial time series and construct financial network which has never seen before. We also did entropic analysis of the financial system using feature matrix which may be used as an early warning of the financial market crash. So, the feature ranking method is a novel approach in financial system.

One of the core techniques of machine learning is supervised learning^18^. In this method, the model tries to learn from a given dataset. In the dataset, there consists a dependent variable which is the target and a set of independent variables which are the features. Often, all the features don’t influence the target with same degree. Generally, the target depends more on some features than some others. Taking this into account, we can rank the features according to their influence on the target. In machine learning, this phenomenon is known as feature ranking^19^. There are different methods such as Random Forest^20^, ReliefF^21^, Decision Tree^22^, Gradient Boosting^23^, which calculate feature ranking implicitly. As features with low ranks usually have a very little or no influence on the target variable, it doesn’t contribute to the accuracy of the machine learning model^15^. Rather they complicated the model yet causing a major degrading called over fitting. Hence, we can improve any machine learning model by simply discarding the insignificant features. We can use this whole process of modern complex network reconstruction method to identify the backend structure of stock markets.

Now, we will introduce how we apply the feature ranking approach on empirical time series. We consider every company as an individual dynamic system and monitor them, which in turn generate a time series. We assume the structure of the studied network to be hidden in a black box and try to reconstruct it using the time series provided by all the stocks individually. As we see, different analytic tools are available in order to reveal the black box. Among those tools, machine learning approaches seem more promising. In this paper, we propose our method of reconstructing networks for stock markets from discrete time series by applying the Feature Ranking^19^, which has never been used with the financial time series data before. Here we use the monitored discrete time series data to measure how much the target is influenced by each feature and compute the feature ranking accordingly. Some features have such strong impact on the target that we can safely assume that those features have some connection edges with the target node^15^. We use logarithmic return ${r}_{t}$ of the closing prices of stocks of the current day to predict the state or return ${r}_{t+1}$ of the target stock in the next day. While predicting ${r}_{t+1}$, the importance of stocks in predicting the state of the target stock is calculated by the Random Forest model^20^. After applying the prediction model on the whole dataset $D$, we find the importance of all stocks in predicting the state of all other stocks. In this way, the feature importance matrix or feature ranking matrix $F$ is calculated where stocks act as features. Here, the feature ranking matrix shows asymmetric properties which makes sense because a powerful stock company may have influence on a relatively less powerful company but vice-versa may not applicable. Hence, we represent a directed network in this paper. We calculate both static and dynamic threshold which gives us two different networks for each year. We then analyze the network using different topological properties. Some properties e.g. average clustering coefficient^24^, Shannon entropy^25^ shows good result for both static and dynamic network and capable of identifying crisis moments where other properties obtain good result on static network only. We also show the most influential companies during crisis moments. We show that, feature ranking approach infer more accurate network structure for financial market than existing methods.

## Results

**Figure 1:**
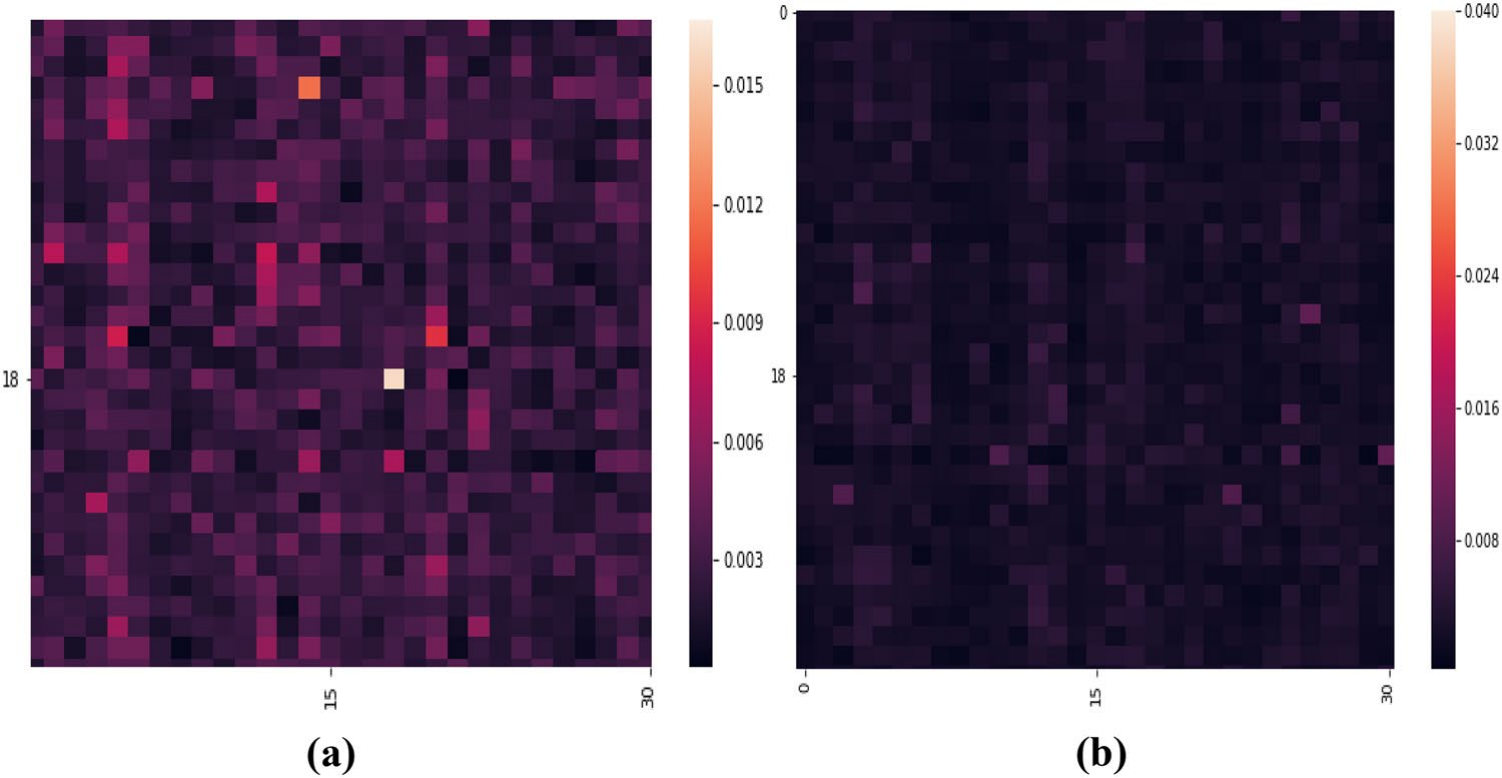
Feature ranking matrices of 30 companies out of 375 companies: (**a**) 2008 (**b**) 2011. Here, we chose 30 companies to show finer map. The light shaded color in heat map indicates the companies which influence more to other companies. The influences of companies during European sovereign debt crisis were higher than global financial crisis.

We calculate feature ranking matrix based on the machine learning models. The feature ranking matrix measures feature importance in terms of probability. We choose Random Forest which uses Gini Impurity or Information Gain approach which finds the best split. It calculates Gini feature importance implicitly which is nothing but the probability of contribution in prediction. Figure 1 shows the feature ranking matrices for two different crises 2008 and 2011 as shown in Figure 1a and b, respectively. The matrices are asymmetric which means unidirectional relationship between two stocks as giant stocks may influence most other stocks but may not be affected much by them all.

**Figure 2:**
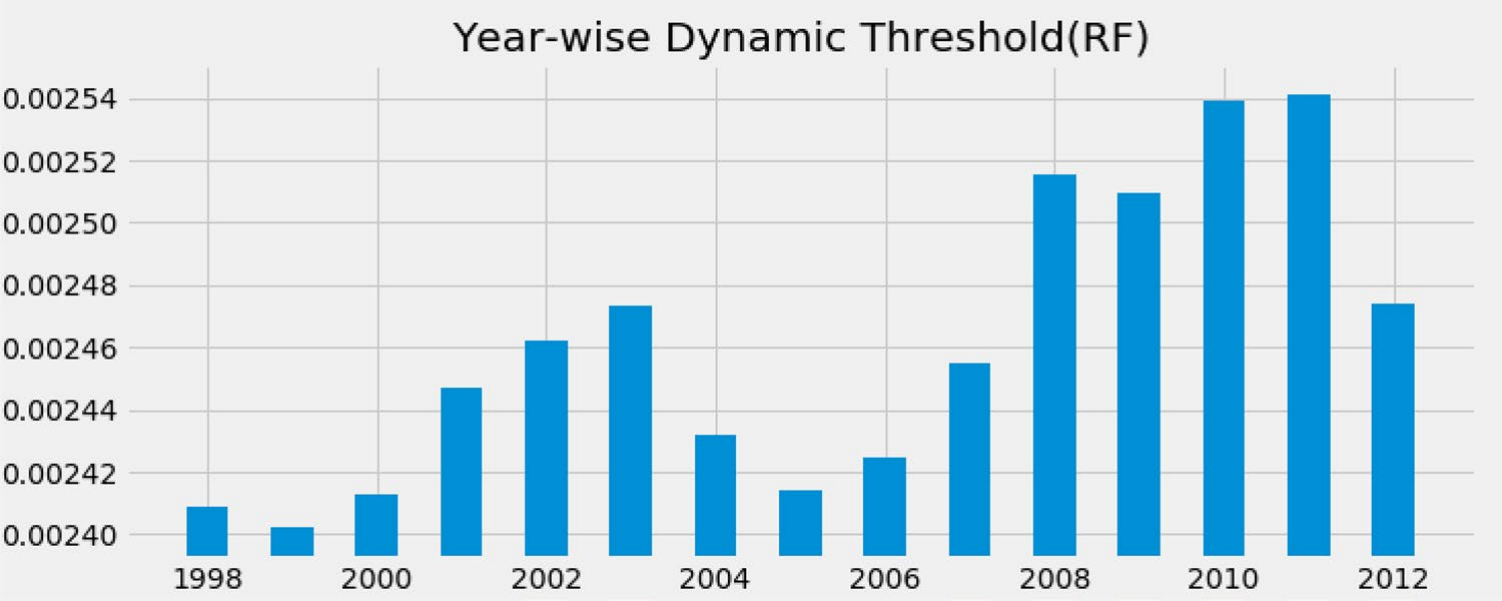
Fluctuation of dynamic threshold over different monitoring years.

We calculate the dynamic threshold and static threshold from elements of the ranking matrix. Figure 2 shows how dynamic thresholds changes with one-year time window. During different crisis years, the dynamic threshold changes to higher values than any normal period. The trend shows that the threshold remains smaller in the beginning of the ‘dot-com bubble’ (2000) and gradually increased up to 2003. The dynamic threshold acts as a good indicator for both the 2008 global finnacial crisis and 2011 European sovereign debt crisis as it holds peak values during these times. On the other hand, static threshold retains its value to $0.0026$. As dynamic threshold assigned high values during crises, the finer network structure and topological properties are not seen. But as static threshold retains its value, it shows proper topological properties.

## Topological properties

**Figure 3:**
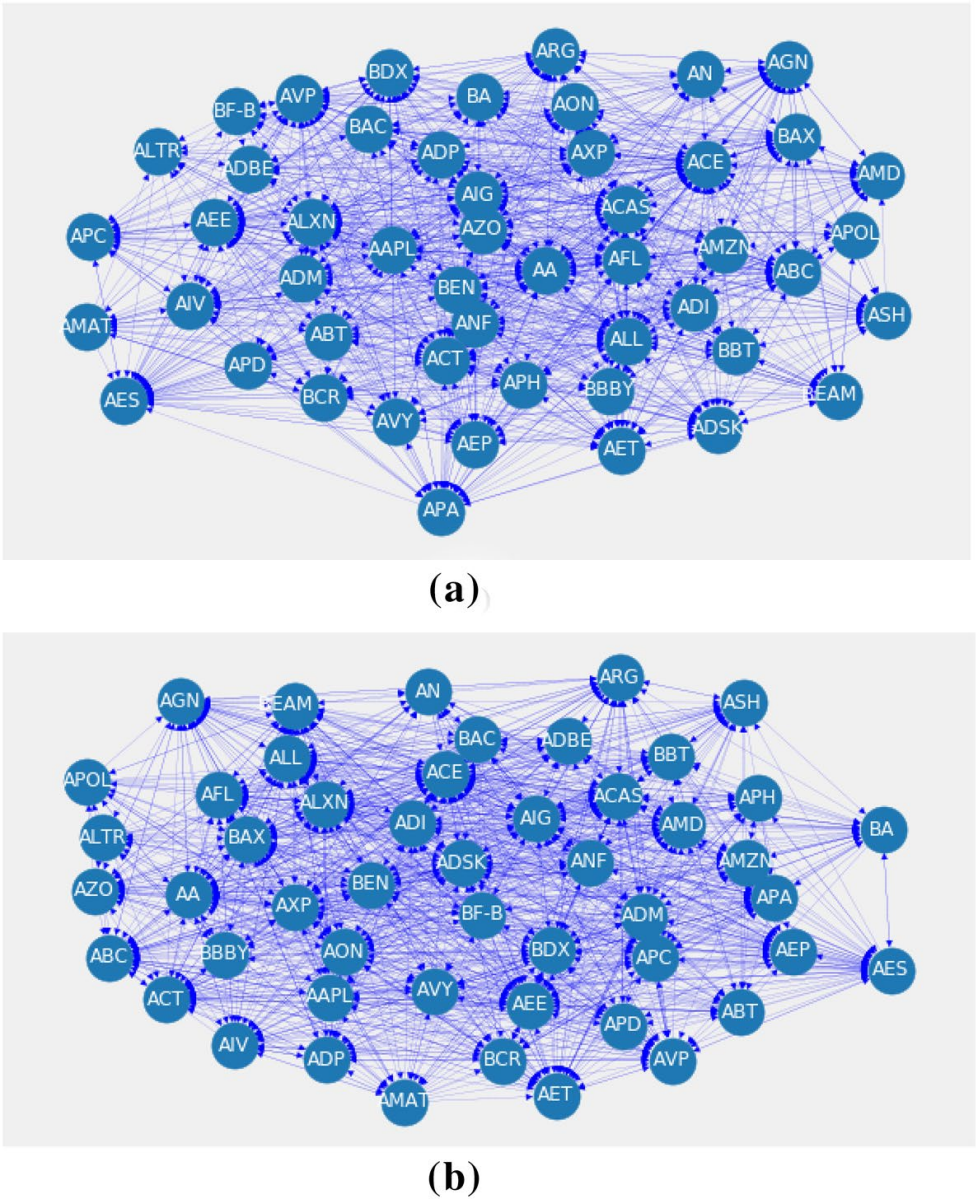
Visualization of directed network structure of 2008 for 50 stocks with: (**a**) Static threshold (**b**) Dynamic threshold. Here, we work on static threshold since it can identify financial crises properly. The higher indegree of a node imply the more influential company in the network. The names of the stock are given in appendix A.

Let’s consider the topological change of the financial network induced by the feature rank matrix**.** In a financial system of size $N$, the companies are known as the nodes of the network and set of links among the nodes depend on threshold. In this paper, the financial networks are constructed from feature ranking matrix *F* assigning a certain threshold $\theta$. Here, we choose two static thresholds $\theta =0.0026$ and $0.0027.$ One is mean value of *F* and another is near to mean value. Since, network is sensitive to the threshold value and for this reason, we took another threshold near mean of the feature ranking matrix. An edge will be added in the financial network if a feature importance is higher than the pre-determined threshold, that is, if ${F}_{ij}>\theta$, where $i$,$j$ = 1, 2…,$N$. We represent the network with directed graph in Fig. 3**for 30 companies**. The network with dynamic threshold shown in Fig. 3b is densely connected than the network generated using static threshold in Fig. 3a. The threshold networks show heavy connections for static and dynamic threshold. The network does not show the scale-free behavior for the degree distribution.

### Average clustering coefficient

The dynamics of average clustering coefficient of the threshold network will be measured over time. The average clustering coefficient is a measure of the compactness and robustness of a network. The clustering coefficient of a vertex $i$ can be expressed as^26–28^,

1 $${C}_{i}=\frac{{m}_{i}}{{n}_{i}({n}_{i}-1)}$$

where ${n}_{i}$ denotes the number of neighbors of vertex $i$, and ${m}_{i}$ is the number of the edges existing between the neighbors of vertex $i$. ${C}_{i}$ is equivalent to 0 if ${n}_{i}$≤ 2. The average clustering coefficient at a specific threshold for the entire network is defined as the average of ${C}_{i}$ over all the nodes in the network, i.e.

2 $$\mathrm{C }=\frac{1}{N}{\sum }_{i=1}^{N}{C}_{i}$$

**Figure 4:**
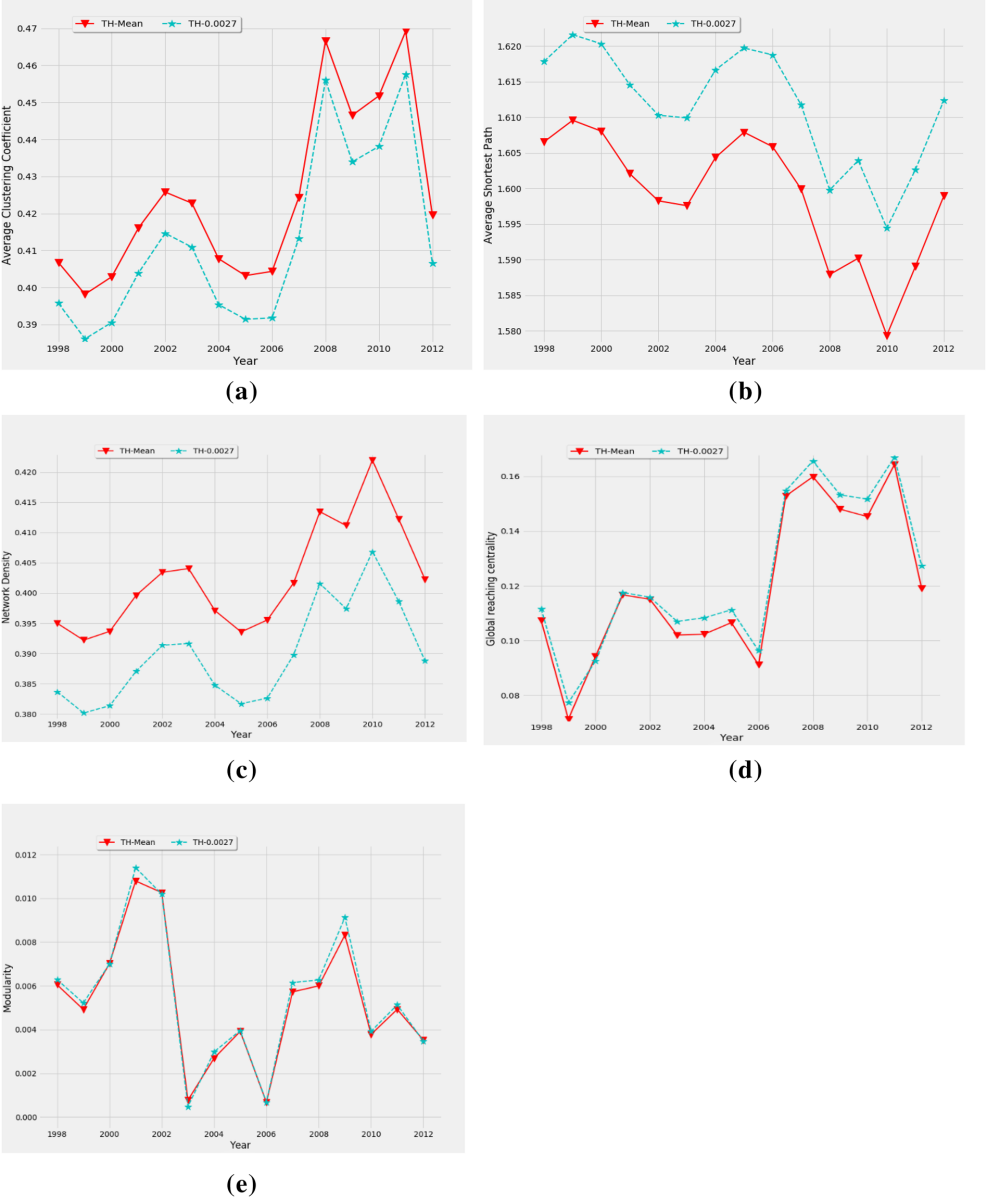
Network properties of the static threshold network for s&p500: (**a**) average clustering coefficient, (**b**) average shortest path length, (**c**) network density, (**d**) global reaching centrality, and (**e**) modularity.

The average culstering coefficient of the financial threshold network of s&p500 is shown at mean threshold $\theta =0.0026$ and $0.0027$ and it remains fixed in all time windows. The samll change of threshold show the sharp change of the network structure and for this reason, we chose another threshold around mean. However, the trend is almost similar. The peak values of average clustering coefficent is found in three crises which are dot-com bubble in 2002, global finnacial crisis in 2008 and European sovereign debt crisis in 2011 shown in dotted line in Fig. 4a. The higher values of average clustering coeffiicent imply that the influence of one company to other is higher during crises. The influence of stocks during global and European crises is higher than dot-com bubble. Since, dot-com bubble hit to the technological companies, the lower average clustering coefficient in this period is appropriate.

### Average shortest path

The average shortest path length of the threshold network is determined with the evolution of time. The characteristic path length or the average shortest pathlength in a cluster can be expressed as^26–28^,

3 $$ \overline{l} = \frac{1}{{N\left( {N - 1} \right)}}\sum\limits_{i,j} {l_{ij} } $$

where ${l}_{ij}$ is the shortest path length between nodes $i$ and $j$.The average shortest path length of the financial threshold network of s&p500 at threshold $\theta =0.0026$ and $\theta =0.0027$ is shown in Fig. 4b. The curve shows a comparatively higher path length in 1999 which indicate that nodes are loosely connected with each other in this year. Then it shows a decreasing trend up to 2003, which implies that nodes become closer during dot-com bubble. The lowest mean shortest path length was observed during ESD crisis (2010) which implies that the dependencies of companies with each other are higher in ESD crisis than global crisis in 2008. On other times, higher mean shortest path length is observed that implies, companies are less dependent to each other and indicates steady state of the market. We also observe that the higher the threshold, the bigger the shortest path is.

### Network density

The network density is the ratio of the number of existing links to the maximum number of possible links, which can be determined as^26,29^,

4 $$\rho =\frac{M}{[N(N-1)]}$$

where $N$ is the total number of the nodes and $M$ is the number of connecting links. The network density of the threshold network of s&p500 at threshold $\theta =0.0026$ and $\theta =0.0027$ is shown in Fig. 4c. At the beginning i.e. at 1998, the curve starts with a higher density indicating the effect of Asian and Russian financial crisis in 1997–1998. The local market also shows a higher trend in 2000–2003 due to the effects of dot-com bubble and September 11 attack, which then declined up to 2005. After that, the highest densities are observed during the mortgage crisis (2007), global crisis (2008) and ESD crisis (2010 and 2011), which indicates a tightly coupled and highly influential network during crisis. The higher value of network density is found in 2010 than global crisis as like average shortest path. During normal period, the curve shows lower density, indicating a loosely connected network while the market is in its calm state.

### Global reaching centrality

The global reaching centrality (GRC) is a global network quantity that calculates the flow of hierarchy of a complex network. It can be defined as^30^,

5 $$GRC=\frac{\sum_{i\in V}[{C}^{max}-C(i)]}{N-1}$$

where $C(i)$ is the local reaching centrality ($LRC$) of node $i$ and ${C}^{max}$ is the maximum value of $LRC$.The global reaching centrality of financial threshold network of s&p500 at threshold $\theta =0.0026$ and $\theta =0.0027$ is shown in Fig. 4d. The curve shows high value during all finnacial crisies from 1998 to 2012. The high values indicate maximal heterogeneous distribution of the $LRC$ which implies maximal hierarchical state of the market during crisis. The sharp change of GRC is observed during subprime mortgage crisis in 2007 which indicate that the influential nodes are placed in the center of the network. This hierarchical state of the market sustains up to 2011 and the market comes back again in low hierarchical state during 2012.

### Modularity

The true community structure in a network which can be quantified by using the phenomenon known as modularity ${Q}_{N}$ can be expressed as^31,32^,

6 $${Q}_{N}=\frac{1}{2m}\sum_{i,j}\left({A}_{ij}-\frac{{k}_{i}{k}_{j}}{2m}\right){S}_{ij}$$

where ${k}_{i}$ is the number of edges of node $i$, and $m=\frac{1}{2}\sum_{i}{k}_{i}$ is the total number of edges in the network, then the probability that two nodes $i$ and $j$ are connected by chance is $\frac{{k}_{i}{k}_{j}}{2m}$. $A$ is the adjacency matrix, entries are in such a way that ${A}_{ij}$ = 1 if node $i$ connects to node $j$ and ${A}_{ij}$= 0 otherwise. $S$ is the modularity matrix; entries are in such a way that ${S}_{ij}$ = 1 if nodes $i$ and $j$ belong to the same module, and zero otherwise. Here, $S$ is calculated in such a way that $i$ and $j$ will belong to the same module if they are of the same type companies. The modularity of financial threshold network of s&p500 at threshold mentioned above is shown in Fig. 4e. As like other network parameter, the higher value of modularity is also found in all crises. In our analysis period, the higher modularity is found during dot-com bubble than other crises. Since this crisis was on the technological companies, the intra-community communication of other groups is incresed because they didn’t want to communicate with the technogial companies. It indicates that intra-module communication increases and inter-module communication falls during crisis times. That is, same type companies depend more on each other during crisis.

### Betweenness centrality

Betweenness centrality is the measurement that captures how much a given node is in-between others. The betweenness centrality can be defined as follows^33^,

7 $$ B\left( u \right) = \mathop \sum \limits_{u \ne v \ne w} \frac{{\sigma_{v,w} \left( u \right)}}{{\sigma_{v,w} }} $$

where ${\sigma }_{v,w}(u)$ is the number of shortest paths (between any couple of nodes in the graphs, here node $v$ and $w$) that passes through the target node $u$. ${\sigma }_{v,w}$ is the total number of shortest paths existing between any couple of nodes (here node $v$ and $w$) of the graph.

Table 1 shows a comparison between top in-between central nodes of the largest subset network and top influential nodes i.e. nodes having most influential edges of two crisis years 2008 and 2011. It also shows the percentage of the number of influential links which is the ratio of number of influential edges of a node and maximal possible influential links. This comparison shows the companies that have most impact on the market during global market crisis in 2008 and ESD crisis in 2011. We found the same companies as central node of the network and the node that have the greatest number of influential edges during two severe crises given in Table 1. During global financial crisis, the Diamond Offshore Drilling and ACE limited were the most powerful companies and they are in the sector of energy and financial services respectively. However, the most leading companies during ESD crisis were Century Link and Sprint Nextel Corporation and they are in the sector of communication services. In this process, we can identify the most powerful companies of the market in any time which can be useful for risk management and portfolio investment.

**Table 1:** Top 2 central and most influential stocks of the year 2008 and 2011 of s&p500.

Top betweenness centrality			Year	Top influential nodes		
Company name	Company type	Percentage of links		Company name	Company type	Percentage of links
Diamond offshore drilling, Inc	Energy	94.4	2008	Diamond offshore drilling, Inc	Energy	94.4
ACE limited	Financial Services	91.7		ACE Limited	Financial Services	91.7
CenturyLink, Inc	Communication Services	95.7	2011	CenturyLink, Inc	Communication Services	95.7
Sprint Nextel Corp	Communication Services	94.1		Sprint Nextel Corp	Communication Services	94.1

The energy and financial services companies were the most leading in the market during global financial crisis while communication services companies were in ESD crisis.

### Entropy

The feature ranking is nothing but the probability of contribution of a company in predicting the return of the target company. The probability of contribution is distributed among 375 companies and the companies which have higher probability imply the most influential in predicting the return of the target. Since total probability of all companies is one, we can calculate the entropy to understand the state of the market. The entropy is calculated as^25^,

8 $$ S = - \frac{1}{N}\mathop \sum \limits_{i,j = 1}^{N} F_{ij} log_{2} \left( {F_{ij} } \right) $$

**Figure 5:**
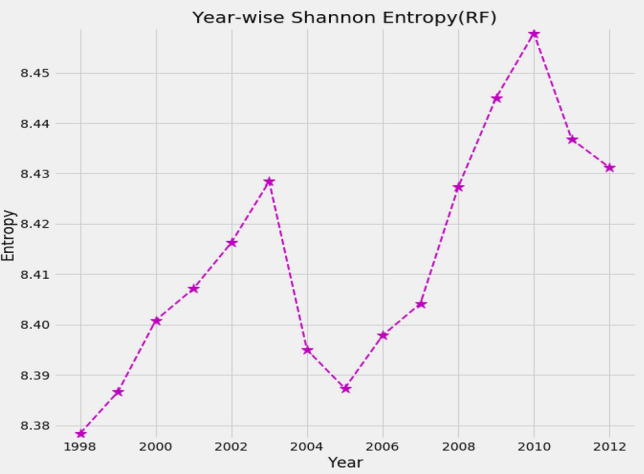
The change of entropy over time. The higher the entropy, the higher the risk of the market.

**Figure 6:**
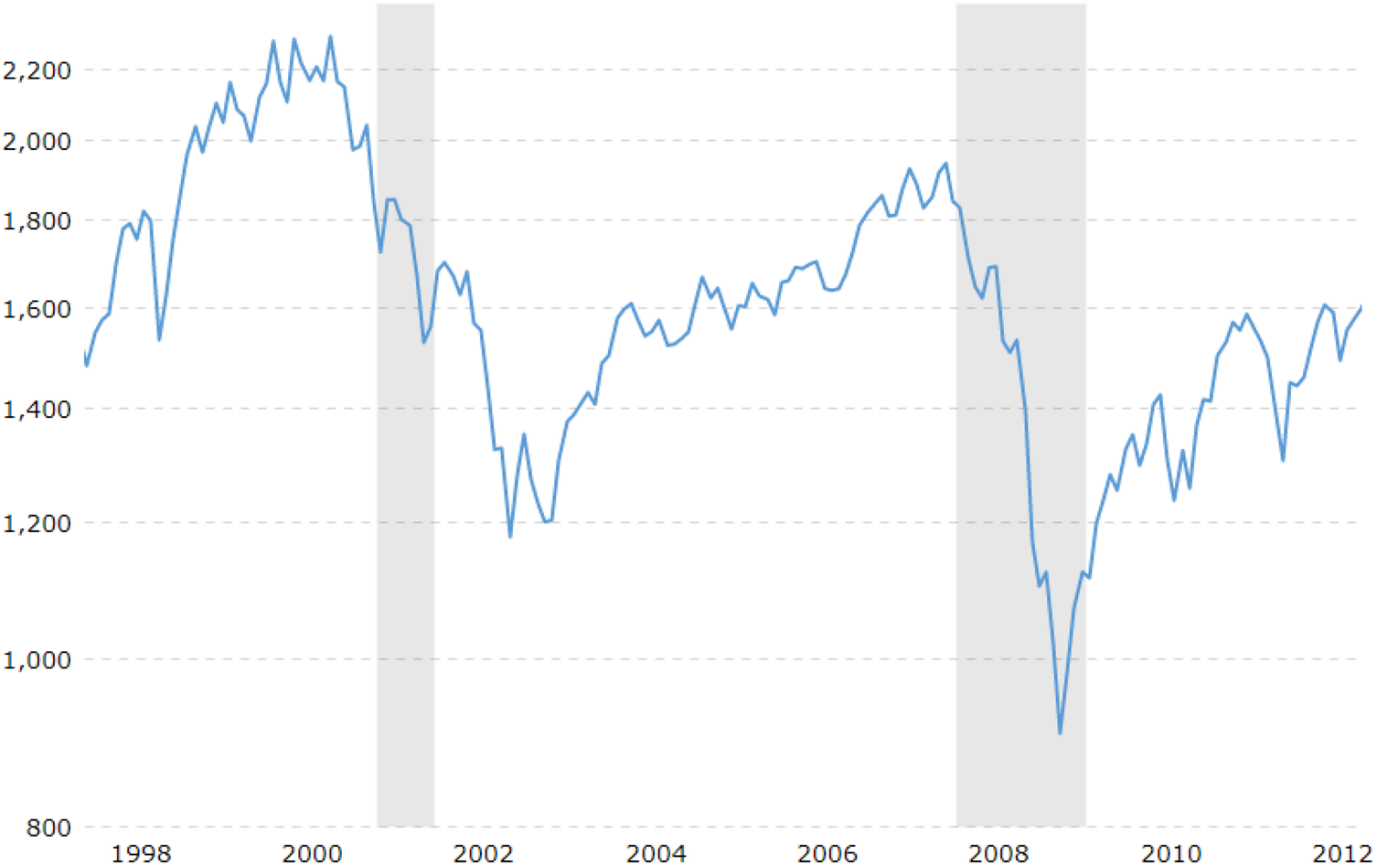
Historical records^35^ of the S&P 500, where the dataset starts from 1998 to 2012. The shaded area indicates the financial crises.

The higher entropy implies that the influences of the companies are becoming equal. We observe that the entropy is increasing from 1998 to 2003 as shown in Figure 5. It means that the market is going to unstable state. We show the index of S&P 500 in the analyzing period in Figure 6. We can compare the change of entropy and the evolution of the index in Figures 5 and 6. Similarly, before global and ESD crises, the entropy is rising and consequently, market falls in crisis. The rise of entropy over time can be used as an indicator of upcoming crisis.

## Discussion

We analyze the daily time series of financial indices using a new approach known as feature ranking. This technique is different than the existing linear and non-linear correlation. The feature ranking is non-linear multivariate technique where multiple features are used to construct feature ranking matrix. We construct threshold network assigning a threshold from the mean of feature matrix and the network topologies are investigated over time. The dynamic change of network properties can identify the financial crises which reflect the crucial state of the market. We identify the companies which are more influential during severe state of the market. We identify four powerful companies belonging to the sector of energy, financial and communication services in two big crises. These companies may shield to protect the crisis. Finally, the entropy estimates and it is seen to increase over time before market crash. The higher entropy can be an indicator for unrest state of the market. Our technique can be an efficient way to analyze the financial time series for development of the market. In future, we will apply this technique in other market using recent time series to monitor the state of the market.

## Methods

### Data analysis

We monitored the daily closing prices for 375 companies listed in the S&P 500 from 1998 to 2012. The data were collected from Yahoo Finance^34^. We then segmented the data using 1-year time window. During this period, the market has covered by different major crises such as ‘dot-com’ bubble in 2000, September11 attack in 2001, the crash of 2002, sub-prime mortgage crisis in 2007, Global financial crisis in 2008 and European sovereign debt (ESD) crisis in 2010 and 2011 as shown in Fig. 6.

### Logarithmic return

The daily return of ${i}$th stock index on day $t$, ${r}_{i}\left(t\right)$, can be defined as,

9 $${r}_{i}\left(t\right)=ln\left[{I}_{i}\left(t\right)\right]-ln[{I}_{i}(t-1)]$$

where ${I}_{i}\left(t\right)$ is the closing price of a stock index $i$ on day $t$. Thus, we can observe and measure the time series of the dynamics of companies.

### Feature ranking approach

Feature ranking is mostly a concept of Machine Learning. Machine learning studies algorithms which learn to make decisions or predictions from some given sample dataset, known as “training data”. These algorithms improve their performance through “experience”^36^. For example, by observing many sample pictures of cat and dog, machine learning algorithm finds distinguishable patterns by which it learns to classify new dog or cat that was never seen. Machine learning is a data driven learning approach which can be used in the circumstances where human knowledge about the studied sector is limited. This is the core reason for which machine learning is widely used in various scientific disciplines, ranging from natural language processing^37^, medicine and biology^38,39^ to financial market analysis^40^, image identification and classification^41^. Machine learning approaches are further divided into some categories on the basis of attributes and feedbacks available to the learning process. Supervised learning is one of such categories where training data consists of both input or independent variables and desired output label or dependent variable. A supervised learning algorithm analyzes the training input data and predicts corresponding output data which get compared with the labeled output data. The model penalized with some feedback value if any mismatch happens between predicted output and labeled output through which the model learns to predict more accurately. Here the input data is said to be the “feature” and the labeled output is called the “target”. Hence, data in supervised learning is given in the feature-target representation. While predicting the target, all the features don’t contribute with the same degree. Some features have more influence on target than some other while predicting the target. Hence, we can determine the importance of features or calculate feature ranks with the help of supervised learning. In this work we adopt feature ranking method introduced by Ref.^15^. We use regression model such as Random Forest^20^ and XGBoost^23^. Both of them are Decision Tree based ensemble Machine Learning algorithm. This type of algorithms make prediction by recursively splitting or partitioning data on the basis of selecting attribute or feature. To find the best split of data, it uses method like Gini Impurity or Information Gain while selecting feature in each step. These methods also measure importance of features in the best split. For example, Gini Impurity calculates Gini feature importance implicitly which is nothing but the probability of contribution in prediction. Feature gets higher importance in prediction if selecting it led to a greater reduction in Gini Impurity. We use these techniques of calculating feature importance in our dataset. In every iteration, we select a node as target and calculate importance of all feature nodes ${F}_{i}$ in predicting the target node, where $i$ is the feature. After all iteration, we find the feature importance for all target nodes and this gives us the feature ranking matrix $F$. The matrix element ${F}_{ij}$ indicates the importance of the feature $j$ in predicting target $i$. The implementation detail of calculating feature ranking matrix is discussed in the following section.

We introduce the method to obtain the matrix element ${F}_{ij}$ through Eqs. (10)–(12). We input the training data set ${D}_{i}$ to the Machine Learning algorithm to obtain the feature element ${F}_{ij}$. Therefore the returns of the index $j$ at the previous time $t$ influence to the feature of the index $i$ at time $t+1$, which represents the element of the feature matrix ${F}_{ij}$. From this feature matrix, we generate the directed networks showing the influence from the source node to target node.

### Reconstruction method

We now briefly dive into our reconstruction method. In the above section, we explain how feature ranking method works. This method can effectively be applied to the problem of reconstructing a dynamical network of stock market from the monitored time series data of its node dynamics (generated using Eq. 9). We select a node (share company) of a dynamical network (stock market) and assume its state as the target, keeping all other node’s dynamics as features to calculate their influence on the selected node. Now from here, we define a supervised learning model e.g. a regression model for predicting the state of the selected node (target company) from the dynamics of all the other nodes in the network. Important fact to note here that, our goal here isn’t to build a predictive model, rather, we only try to construct the feature ranking matrix i.e. our main goal is to rank the importance of the other nodes to the selected one, because a highly ranked node is more likely to be connected to the selected node. We now only need to repeat this procedure for all the stock companies and we can reconstruct the entire network structure of the stock market i.e. which stock company is going to affect a particular stock company’s state.

Let’s consider ${r}_{i}\left(t+1\right)$ at time $t+1$. Its dynamics is influenced by the previous state represented as,

10 $$ r_{i} \left( {t + 1} \right) = f_{i} \left( {r_{1} \left( t \right),r_{2} \left( t \right), \ldots ,r_{N} \left( t \right)} \right), i = 1, \ldots , N $$

where the interaction function ${f}_{i}$ is unknown which can be modeled using the observed time series data and $N=375$. We construct our training dataset ${D}_{i}$ with $L-1$ instance from the monitored time series as,

11 $$ D_{i} = \bigcup\limits_{t = 1}^{L - 1} {(r_{1} \left( t \right),r_{2} \left( t \right), \ldots , r_{N} \left( t \right); r_{i} \left( {t + 1} \right))} $$

where ${r}_{i}\left(t+1\right)$ is our target index and the states of all the other indices at some prior time $t$ is considered as feature index.

In this paper, we don’t model the interaction function $f$, rather we will only focus on network structure of the stock market. To calculate the feature ranking of share companies, we consider the two mentioned algorithms, Random Forest^20^ and XGBoost^23^. By applying any of the above-mentioned feature ranking algorithm $R$ to the training data ${D}_{i}$,we get feature ranks for node $i$,

12 $$\left({F}_{i1},{F}_{i2},\dots ,{F}_{iN}\right)=R\left({D}_{i}\right)$$

where ${F}_{ij}$ tells us the estimated impact of node $j$ (share company $j$) on node $i$ (share company $i$). By estimating this feature importance for all *N* share companies, we construct a feature ranking matrix $F$ of dimension $N\times N$,

13 $$ F = \left( {\begin{array}{*{20}c} {\begin{array}{*{20}c} {F_{11} } & {F_{12} } \\ \end{array} } \\ {\begin{array}{*{20}c} {\begin{array}{*{20}c} {F_{21} } \\ \vdots \\ {F_{N1} } \\ \end{array} } & {\begin{array}{*{20}c} {F_{22} } \\ \\ \cdots \\ \end{array} } \\ \end{array} } \\ \end{array} \begin{array}{*{20}c} {\begin{array}{*{20}c} \cdots & {F_{1N} } \\ \end{array} } \\ {\begin{array}{*{20}c} {\begin{array}{*{20}c} \\ \ddots \\ \\ \end{array} } & {\begin{array}{*{20}c} \vdots \\ \\ {F_{NN} } \\ \end{array} } \\ \end{array} } \\ \end{array} } \right) $$

We see that, the estimated feature ranking matrix shows asymmetric properties i.e. ${F}_{ij}$ may not equal to ${F}_{ji}$. Hence, a company may influence some other companies but vice-versa may not applicable. This makes sense as powerful companies may have unidirectional influence. Hence, here we represent a directed network. We can simply assume that higher the value of ${F}_{ij}$ is, more likely it is that the link $i\to j$ exists.

Finally, we come up with our desired reconstructed adjacency matrix $\widehat{A}$ simply by filtering out low ranked features by setting up a threshold value $\theta$. We calculate both dynamic thresholds and static threshold. In dynamic thresholding, threshold value may change every year depending on the values of $F$, while static threshold retains its value each year. Dynamic threshold ${\theta }_{D}$ can be defined as,

14 $$ \theta_{D} = \frac{1}{N}\mathop \sum \limits_{i = 1}^{N} \left\{ {\frac{{\left( {N + 1} \right)}}{2}} \right\}_{{F_{iJ} }} $$

where ${\theta }_{D}$ is a vector of per year threshold and $N$ is the number of nodes. Here, ${\left\{\frac{(N+1)}{2}\right\}}_{{F}_{iJ}}$ is the median of row $i$ in $F$ where $J$ indicates $N$ columns and ${\left\{\frac{(N+1)}{2}\right\}}$ is the index of the median in row $i$. On the other hand, static threshold can be defined as,

15 $${\theta }_{S}=\frac{1}{N*N}\sum_{i,j=1}^{N}{F}_{ij}$$

where ${F}_{ij}$ is an entry of row $i$ and column $j$ of the feature ranking matrix. We then calculate the reconstructed adjacency matrix $\widehat{A}$ from the feature ranking matrix $F$ with the help of the estimated thresholds as follows,

16 $$ \hat{A}_{ij} = \left\{ {\begin{array}{*{20}c} 0 & {if F_{ij} \le \theta } \\ 1 & {if F_{ij} > \theta } \\ \end{array} } \right. $$

## Supplementary Information

Supplementary Information.
