# Supplementary material for: Structure and dynamics of financial networks by feature ranking method
Source: Sci Rep. 2021 Sep 2;11:17618. doi: 10.1038/s41598-021-97100-1 (PMC8413283; doi:10.1038/s41598-021-97100-1)
Supplement: Supplementary file 1 — Supplementary Information. [file 41598_2021_97100_MOESM1_ESM.docx]

# **Appendix A**

Table A1. Names of the companies used in threshold networks

| **Number** | **Abbreviation** | **Full Name** |
| --- | --- | --- |
| 1 | AA | Alcoa Corporation |
| 2 | AAPL | Apple Inc. |
| 3 | ABC | AmerisourceBergen Corporation |
| 4 | ABT | Abbott Laboratories |
| 5 | ACAS | American Capital Ltd |
| 6 | ACE | ACE Limited |
| 7 | ACT | Advisor Shares Vice ETF |
| 8 | ADBE | Adobe Inc |
| 9 | ADI | Analog Devices, Inc. |
| 10 | ADM | Archer-Daniels-Midland Company |
| 11 | ADP | Automatic Data Processing, Inc. |
| 12 | ADSK | Autodesk, Inc. |
| 13 | AEE | Ameren Corporation |
| 14 | AEP | American Electric Power |
| 15 | AES | AES Corp |
| 16 | AET | Aetna Inc |
| 17 | AFL | AFLAC Inc |
| 18 | AGN | Allergan, Plc |
| 19 | AIG | American International Group, Inc. |
| 20 | AIV | Apartment Investment and Management Company |
| 21 | ALL | The Allstate Corporation |
| 22 | ALTR | Altera Corp |
| 23 | ALXN | Alexion Pharmaceuticals, Inc. |
| 24 | AMAT | Applied Materials, Inc. |
| 25 | AMD | Advanced Micro Devices, Inc. |
| 26 | AMZN | Amazon.com, Inc. |
| 27 | AN | AutoNation, Inc. |
| 28 | ANF | Abercrombie & Fitch Co |
| 29 | AON | Aon plc |
| 30 | APA | Apache Corporation |
| 31 | APC | Anadarko Petroleum Corporation |
| 32 | APD | Air Products and Chemicals, Inc. |
| 33 | APH | Amphenol Corporation |
| 34 | APOL | Apollo Global Management, LLC |
| 35 | ARG | Argos Therapeutics, Inc. |
| 36 | ASH | Ashland Global Holdings Inc. |
| 37 | AVP | Avon Products, Inc. |
| 38 | AVY | Avery Dennison Corporation |
| 39 | AXP | American Express Co |
| 40 | AZO | AutoZone, Inc. |
| 41 | BA | The Boeing Company |
| 42 | BAC | Bank of America Corporation |
| 43 | BAX | Baxter International Inc. |
| 44 | BBBY | Bed Bath & Beyond Inc. |
| 45 | BBT | BB&T Corporation |
| 46 | BCR | Bard (C.R.) Inc. |
| 47 | BDX | Becton, Dickinson and Company |
| 48 | BEAM | Beam Inc. |
| 49 | BEN | Franklin Resources, Inc. |
| 50 | BF-B | Brown-Forman Corporation |
